# Compact genomic architecture of the axolotl MHC region: setting the record straight

**DOI:** 10.1007/s00251-025-01391-x

**Published:** 2025-11-15

**Authors:** Magdalena Migalska, Krzysztof Fluder, Katarzyna Dudek, Wiesław Babik

**Affiliations:** Institute of Environmental Sciences, Faculty of Biology, Jagiellonian University, Kraków, Poland

**Keywords:** Salamanders, Amphibians, *Ambystoma mexicanum*, MHC architecture, Genome annotation, Comparative immunogenomics

## Abstract

The Major Histocompatibility Complex (MHC) is a gene-dense genomic region essential to adaptive immunity, exhibiting both conserved features and lineage-specific rearrangements across jawed vertebrates. Amphibians, as basal tetrapods, offer critical insights into MHC evolution; however, research has largely focused on anurans (e.g., *Xenopus*), while urodeles remain understudied, primarily due to their exceptionally large genomes. With recent advances in sequencing technologies, chromosome-scale assemblies for urodeles are now becoming available, opening new opportunities to explore their MHC architecture. Yet, the MHC region remains notoriously challenging to annotate due to its complexity, high polymorphism, and dynamic evolutionary history. For instance, the initial annotation of the axolotl (*Ambystoma mexicanum*) MHC relied heavily on synteny with mammalian genomes, which led to an overestimation of its size and misinterpretation of its structure. These inaccuracies have fueled evolutionary debates regarding the ancestral genomic structure of the MHC. Here, we present a comprehensive re-annotation of the axolotl MHC, revealing a typical organization found in tetrapods other than eutherian mammals: core MHC region with several, expressed MHC class I genes, tightly linked to their antigen processing genes, and to single loci of MHC class II genes. Contrary to the previous report, class I and class II genes are not separated by class III genes, and the overall MHC region is relatively compact (by axolotl genome standards). These findings correct earlier misconceptions and emphasize the need for annotation strategies that reflect the complex and lineage-specific nature of genomic regions rich in immune genes.

**Supplementary Information:**

The online version contains supplementary material available at 10.1007/s00251-025-01391-x.

## Introduction

The Major Histocompatibility Complex (MHC) is a cornerstone of the adaptive immune system in jawed vertebrates. MHC genes are located within a complex, gene-dense genomic region that exhibits several well-conserved features, but also major rearrangements across different vertebrate groups (Kulski et al. 2002). Among these, amphibians occupy a key evolutionary position—as early branching tetrapods—which makes them particularly informative for understanding the evolution of MHC architecture. Much of the foundational knowledge about amphibian MHC has been derived from detailed studies of the anuran genus *Xenopus*, where descriptions of genomic organization (notably for *X. laevis* and *X. tropicalis*, (Ohta et al. 2006; Session et al. 2016)) are complemented by a range of genetic and functional immunological studies examining the expression and function of several MHC molecules (reviewed in Lopez Ruiz et al., 2023).

In contrast, the second-largest amphibian order, Urodela, has been far less studied. This gap is largely due to the formidable sizes of urodele genomes (10–120 Gb, Gregory 2025), which have historically precluded genome-scale analysis. However, with the rapid advancement of sequencing technologies and chromosome conformation capture techniques, the barriers to exploring them are finally being overcome. The chromosome-scale assembly of the giant genome of the axolotl, *Ambystoma mexicanum*, (Nowoshilow et al. 2018; Schloissnig et al. 2021; Smith et al. 2019) ushered in a new era for Urodela genomics (Card et al. 2022; Kosch et al. 2024; Pyron et al. 2024).

However, genome assembly is only the first step, which must be followed by the accurate annotation of the MHC region – a task notoriously challenging due to a combination of interrelated factors. First, complex expression patterns (low or restricted to specific tissues or developmental stages) complicate automated annotation, which nowadays relies heavily on RNAseq data (Gabriel et al. 2024). Semi-manual draft annotations, which often use BLAST searches against protein databases, can be misguided by the evolutionary dynamics of the genes involved, with gene birth-and-death process frequently leaving behind numerous pseudogenes and gene fragments. Also, high polymorphism, duplication and intra-specific copy number variation of certain genes can further impede reconstruction (Fawal et al. 2014). Moreover, some gene groups evolve so rapidly that 1:1 orthology is lost even among relatively closely related taxa, impeding coherent naming of detected genes (Hughes et al., 1989). A separate category of difficulties relates to the highly specialized and layered nomenclature used in MHC research, which can appear confusing, if not misleading, to those less familiar with the field. For instance, the term “MHC” may refer strictly to genes encoding *bona fide*, classical MHC molecules that present antigens to T cells, or it can also encompass non-classical or MHC-like molecules with diverse functions but a similar structural fold. It can also refer more broadly to the entire genomic region that contains these genes, alongside dozens of other genes, unrelated to antigen presentation. In fact, all three usages are common and not incorrect.

Classical MHC genes encode two types of cell surface glycoproteins that present peptide antigens to T cells: MHC class I (also referred to as Ia) and MHC class II. Class I molecules are generally ubiquitously expressed and present cytosol-derived antigens to cytotoxic CD8⁺ T cells. In contrast, class II molecules are primarily expressed on antigen-presenting cells and present extracellularly derived peptides to helper CD4⁺ T cells (Punt et al. 2019). Beyond these, a broader definition includes the so-called non-classical MHC genes. Non-classical class I genes, or MHC Ib, are typically non-polymorphic, less ubiquitously expressed, and while some retain antigen-presenting capabilities many have evolved other roles (for a comprehensive review in mammals, see Adams et al., 2013). Non-classical MHC class II molecules, such as DM and DO, generally function as chaperones that assist classical MHC class II molecules (Kulski et al. 2002).

**Fig. 1:**
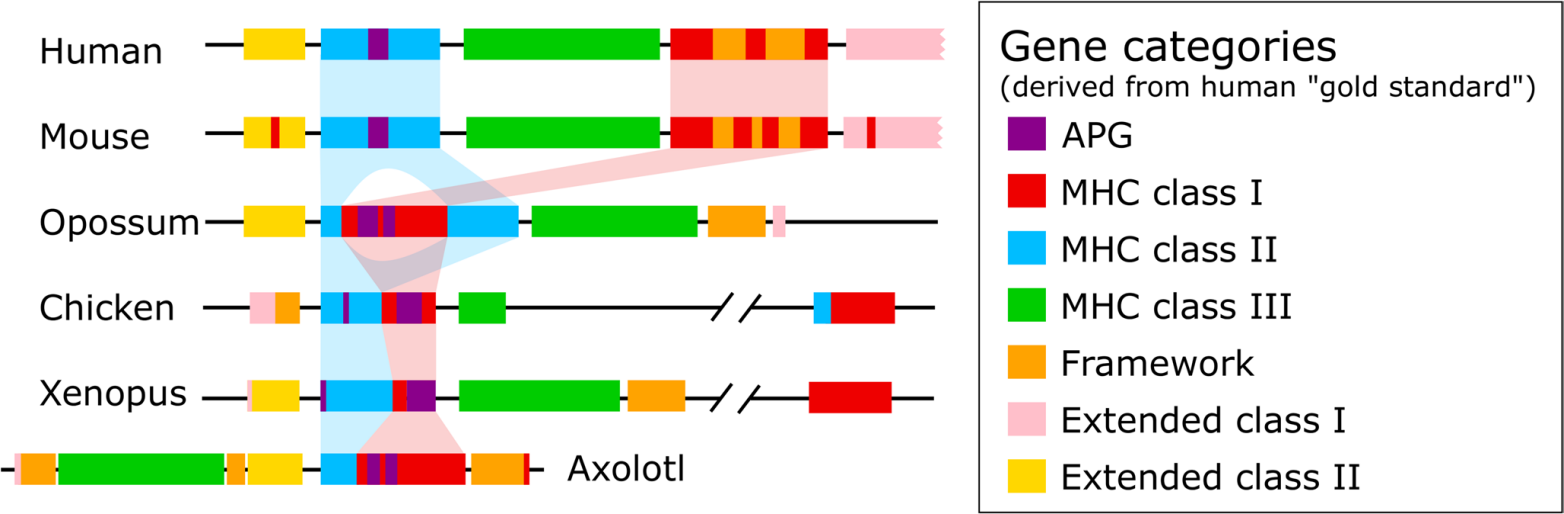
Schematic representation of the genomic MHC organization in tetrapods. Gene blocks are colored according to categories derived from human studies and adapted from (Belov et al. 2006). Based on data from this article (axolotl) and (Belov et al. 2006; Kaufman 2018, 2021; Session et al. 2016). Drawn not to scale.

Adding to the complexity, the term MHC is also used to describe a large genomic region (up to 7.6 Mb in humans, when including the full “extended regions” as defined by Horton et al. 2004). This region includes the classical MHC class Ia and class II genes, several MHC class Ib and non-classical class II genes, as well as dozens of other genes—both immune-related and unrelated. Understandably, the earliest comprehensive genomic studies of the MHC focused on humans (Beck et al. 1999), and while it is now clear that the human MHC structure is derived and specific to eutherian mammals (Fig. 1), it continues to serve as a “gold standard” model for comparative analyses (Kulski et al. 2022).

A characteristic feature of the MHC of placental mammals is the separation of the class I and class II regions by the so-called class III region (centromere-to-telomere order: class II – class III – class I, total length of ca. 3.5 Mb) (Fig. 1). The class I region contains classical class Ia genes and several non-classical class Ib genes (though additional MHC-I-like genes are found on other chromosomes), along with a set of interspersed, conserved, non-immune “framework genes” (Amadou 1999). These framework genes have proven particularly valuable for constructing comparative maps across mammals, because the rapid evolution of MHC class I genes erases orthologous relationships even among closely related species (Hughes et al., 1989). The class III region harbors a diverse array of genes, most of which are not involved in antigen presentation. Instead, they include cytokines and components of the complement system, as well as genes not involved in immune function (Horton et al. 2004). The class II region contains the classical class II genes, non-classical genes (*DM*, *DO*), and a group of antigen-processing genes (hereafter referred to as APGs). APGs (the peptide transporters *TAP1* and *TAP2*, and the immunoproteasome subunits *PSMB8* and *PSMB9*) are essential for preparing peptides for presentation by MHC class Ia molecules (Pishesha et al. 2022). Flanking both sides of the MHC region are the so-called “extended” MHC regions. The extended class II region, which is directly adjacent to the class II region (starting at *COL11A2*), is well defined and contains a conserved block of genes (ca. 0.2 Mb), most of which are not immune-related. Notable exceptions include *TAPBP* (tapasin), a component of the peptide-loading complex for MHC class Ia. The extended class I region, on the opposite end, is rarely described in full in the literature, and is rather accentuated as a region after the last MHC class I gene (*HLA-F*, a class Ib gene), which includes genes such as *MOG* and *GABBR1*. In its entirety, it extends past *HFE* (an MHC-I-like gene), and contains many olfactory receptors, histone genes, and tRNA genes, totaling to up to 3.9 Mb (Horton et al. 2004).

This picture was significantly altered when early studies of non-mammalian vertebrates, such as chicken, *Xenopus*, and nurse sharks, revealed that the MHC organization observed in placental mammals is not representative of all jawed vertebrates (Kaufman et al. 1999; Ohta et al. 2002, 2006). Instead, these findings suggested that the ancestral MHC configuration featured a “true” class I region, where MHC class Ia genes were linked with their associated APGs, and this block was directly adjacent to the class II region (Fig. 1). Together, these formed what is often referred to as the “core” MHC locus, or the combined class I/II region. While the physical linkage between the class I and class II regions was completely lost in teleost fish (Grimholt et al., 2016), the ancestral, core MHC organization appears to have been retained in cartilaginous fish, basal bony fishes (lungfish, bowfin, reedfish) (Thompson et al. 2021; Veríssimo et al. 2023), as well as a range of non-eutherian tetrapods, such as lizards (Card et al. 2022), alligators (Ke et al.), frogs (Ohta et al. 2006; Session et al. 2016), certain birds, like chicken and stork (Kaufman et al. 1999; Tsuji et al. 2017), and some marsupials, like the opossum (Belov et al. 2006).

Furthermore, the tight linkage between MHC class Ia genes and their associated APGs within the core MHC I region was proposed to promote coevolution between these components, while simultaneously constraining the expansion of MHC Ia genes within the core MHC region (reviewed in Kaufman 2015; Ohta et al., 2015). However, the first co-segregation studies in the axolotl (Sammut et al. 1999) confirmed the linkage of class I and II genes, but also suggested the expansion of presumably polymorphic Ia and Ib genes within or in proximity to the core MHC region, thus challenging some of these early assumptions. Two decades later, the release of the first high-quality urodele genome (Nowoshilow et al. 2018; Schloissnig et al. 2021) provided a long-awaited opportunity to examine MHC organization in this group directly. However, rather than offering a resolution, the results added further confusion. The axolotl MHC was suggested to resemble the mammalian configuration, with class III genes separating the class I and class II regions (Schloissnig et al. 2021). This unexpected finding has reignited the debate over key assumptions about amphibian—and more broadly, non-mammalian—MHC evolution (Sabino-Pinto et al., 2025).

However, we contend that the characterization of the MHC locus presented by Schloissnig et al. (2021) was affected by certain methodological oversights, ultimately resulting in a misrepresentation of the extent and structure of the MHC region. Similarly, subsequent analyses of MHC organization in multiple amphibian genomes, including that of the axolotl (He et al. 2023), employed imprecise nomenclature, which further complicated interpretations. As these misconceptions continue to echo through recent literature - both MHC-related (Martin et al., 2022; Sabino-Pinto and Maan, 2025) and also broader, genomic reports (including the presentation of the second-ever, chromosome-scale urodele genome of *Pleurodeles waltl*, Brown et al. 2025) - we believe it is essential to address and correct them.

In this study, we present a comprehensive annotation of the axolotl MHC region, followed by an in-depth discussion of prior interpretive shortcomings. Specifically, we demonstrate that the axolotl exhibits the typical MHC organization found in non-eutherian tetrapods: a core MHC region in which MHC class I genes—including both putative Ia and Ib (as originally suggested by Sammut et al. 1999)—are closely linked to each other, as well as the APGs and MHC class II genes. Contrary to previous reports, MHC class I and class II genes are not separated by class III genes, and the overall MHC region remains relatively compact (by axolotl genome standards), rather than expanded. These findings offer a necessary correction and reaffirm the importance of amphibian models for understanding the evolutionary trajectory of the vertebrate MHC.

## Materials and methods

### Genome assembly

We used the KY_AmexF1_1 genome (NCBI RefSeq: GCA_040938575.1), a maternal pseudohaplotype created from the genome sequence of the F1 female progeny of a cross between a female *A. mexicanum* and male *A. tigrinum*. It was the latest (30 Jul 2024), chromosome-scale genome assembly of the axolotl genome (scaffold N50: 1.5 Gb, contig N50: 23.1 Mb). We note that this is a distinct genome assembly than the one used by Schloissnig et al. (2021) (AmexG_v6.0-DD, scaffold N50: 1.2 Gb, contig N50: 218 kb). However, we confirmed that our findings regarding MHC organization are consistent across both assemblies – see Supplementary Note 1, Figure S1 and S2 for details (Supplementary File 1 ). For subsequent annotation, we extracted chromosome 13, known to possess MHC region in this species (chromosome RefSeq ID: NC_090938.1, length: 848.6 Mb).

### Representative MHC sequences

To obtain full length coding sequences of the axolotl MHC transcripts we used two publicly available PacBio IsoSeq datasets: (i) ca. 165 k HiFi reads from embryo and blastema (Schloissnig et al. 2021), NCBI SRA accession SRR13825941), (ii) ca. 560 k HiFi reads from pooled mRNA of multiple tissues (Qin et al. 2024), Chinese GSA accession (CRR945870). The PacBio HiFi reads from dataset (i) were clustered into transcripts using the SMRT Tools v. 13.1 lima, isoseq refine and isoseq cluster tools. Only high quality (hq) IsoSeq transcripts generated by this pipeline were used in subsequent analyses. The HiFi reads in dataset (ii) were already clustered into transcripts and these transcripts were used in the analyses. MHC protein sequences from the axolotl available in GenBank were used to tblastn (Camacho et al. 2009) search of the database of IsoSeq transcripts from the combined datasets (i) and (ii) at an E-value threshold of 1e-30. TransDecoder v 5.7.1 (Haas 2024) was used to identify Open Reading Frames (ORF) in IsoSeq transcripts (both complete and partial ORFs were allowed, single best ORF per transcript). Visual inspection of the protein alignments indicated that the sequences were often highly similar, differing only in the presence/absence of entire exons. Therefore, to reduce redundancy, we clustered the proteins based on sequence identity. Clustering was performed using the function Clusterize from the R package DECIPHER (Wright 2016) at the protein sequence divergence threshold of 0.1, ignoring regions containing gaps (penalizeGapLetterMatches = FALSE). The longest protein was selected as representative of each cluster.

### MHC region annotation

First, we confirmed the location of the MHC region by blastn searches with representative MHC sequences (obtained as described above) within chromosome 13 only. Then, using the genome annotation file (GCF_040938575.1-RS_2024_10) associated with the genome assembly, we extracted predicted protein sequences (using gffread v 0.12.8, Pertea & Pertea 2020) and re-annotate them with eggNOG-mapper v2 (Cantalapiedra et al. 2021), a tool for functional annotation based on fast orthology assignments using precomputed eggNOG v5.0 clusters and phylogenies (Huerta-Cepas et al. 2019).

Finally, we cross-referenced the genes on axolotl chromosome 13 with the list of genes located in the human MHC region (GRCh38.p14, chr6:28,510,120–33,480,577; Ensembl release 114). To facilitate comparative analysis, we classified these genes into categories defined by Belov et al. (2006): (i) Class I, which includes putative classical or non-classical MHC class I genes; (ii) Class II, which encompass classical MHC class II genes, DM genes, and *BRD2*—a non-MHC gene closely linked to the Class II region in most genomes studied to date; (iii) Antigen Processing Genes (APGs), including *PSMB8*, *PSMB9*, *TAP1*, and *TAP2*; (iv) Extended Class II, spanning from *COL11A2* to *ZBTB9*, and including *TAPBP* (which is predominantly located within the extended Class II region of species examined to date, but is functionally an APG); (v) Extended class I, limited to the proximal 1.1 Mb of the extended class I, corresponding to the portion designated as the MHC region in GRCh38.p14; (vi) Class III, and (vii) Framework genes, which are conserved, non-MHC genes interspersed within the eutherian MHC Class I region (Amadou 1999). In non-placental mammals, framework genes can be adjacent to the class III MHC region, and in non-mammalian genomes they can be more widely dispersed. Genes not found within the human MHC region were not assigned to any category.

We found that the automatic gene prediction and annotation was mostly inaccurate for MHC genes, in particular MHC class I genes, which were either missing, fused, or misidentified as distant MHC-like genes. Therefore, we manually annotated all MHC genes, supplementing and correcting the information from the initial gene models from the automatic prediction with: (i) RNAseq reads from an adult spleen (Illumina, 2 × 100 bp, SRA accession SRR5341570), mapped to the genome with hisat2 v. 2.2.1 (Kim et al. 2019), (ii) representative MHC sequences (obtained as described above), and (iii) IsoSeq transcripts. The latter two were mapped to the genome with minimap2 v 2.28, (Li 2018). All data tracks were visualized using IGV v2.18.4 (Robinson et al. 2011) and coding sequence (CDS) coordinates were identified. Although we did not perform full structural annotation (i.e., including untranslated regions, UTRs), we identified complete CDSs and defined their intron–exon structures. The retrieved CDSs were checked for internal stop codons and are available as Supplementary File 2 associated with this article. An updated annotation file was generated for chromosome 13, containing both automated predictions of non-MHC genes reannotated with eggNOG-mapper (with category assignment), and annotated MHC genes (as described above). This file is available as Supplementary File 3.

### Comparison to previously published axolotl MHC sequences

We compared our genomic sequences with those previously reported sequences for this species (Laurens et al. 2001; Sammut et al. 1999; Tournefier et al. 1998). Earlier studies estimated the number of MHC class I loci based on unique sequences derived from cloned and Sanger-sequenced amplicons. However, these early datasets did not undergo artifact filtering or correction, standards that were later adopted in MHC genotyping (e.g., acceptance of variants supported by multiple independent clones and discarding singletons likely to be sequencing errors; Babik 2010; Lenz and Becker, 2008). As a result, the number of MHC class I loci was likely overestimated. To re-evaluate these data, we re-examined individual clones (GenBank accessions AF156606–AF156614 and U83137–U83138) and generated consensus sequences using EMBOSS Cons (Rice et al. 2000) (available as Supplementary File 4). To assess the relationship between the variants identified by Sammut et al. (1999) and the loci described in our genomic data, we performed a phylogenetic analysis in MEGA v10 (Kumar et al. 2018). For MHC class II, we aligned our full-length MHC *DAB* sequence with the only available record from the literature, a *DAB* sequence reported by Laurens et al. (2001) (GenBank protein ID: AAG42326.1).

### MHC class I expression

To complement the MHC class I gene annotation with expression data, which provides insight into gene functionality and helps distinguish between classical and non-classical genes, we analyzed transcriptome data collected from laboratory-reared axolotls (held at the Institute of Environmental Sciences, Jagiellonian University, Kraków, Poland) at multiple developmental stages and from various tissues. The axolotl is a neotenic species that does not undergo metamorphosis under normal conditions. Its development proceeds gradually: larvae hatch at approximately two weeks post-fertilization and complete limb and digit formation around two to three months of age (though developmental pace varies substantially and depends on factors such as larvae density, food availability, and temperature). Afterwards, individuals continue to grow and undergo minor physiological changes, but they do not experience a dramatic, transformative event comparable to metamorphosis. Instead, they reach sexual maturity at around one year of age while retaining aquatic larval features, such as external gills (Adamson et al. 2022). Details on animal husbandry, tissue collection, library preparation and sequencing are provided in Supplementary Note 2 and Table S1 (Supplementary File 1). Tissues were sourced from one mature female (2.5 years old) and larval stages 51, 53, 56 and 57 (according to Nye et al. 2003 staging), encompasses time-series from approx. 6 to 16 weeks post fertilization. RNA-seq reads were mapped to the genome using hisat2 (Kim et al. 2019) and read counts for each annotated MHC class I locus were obtained with featureCounts v. 2.0.8 (Liao et al. 2014). Expression was estimated as Fragments Per Kilobase of transcript per Million mapped reads (FPKM).

## Results

**Fig. 2:**
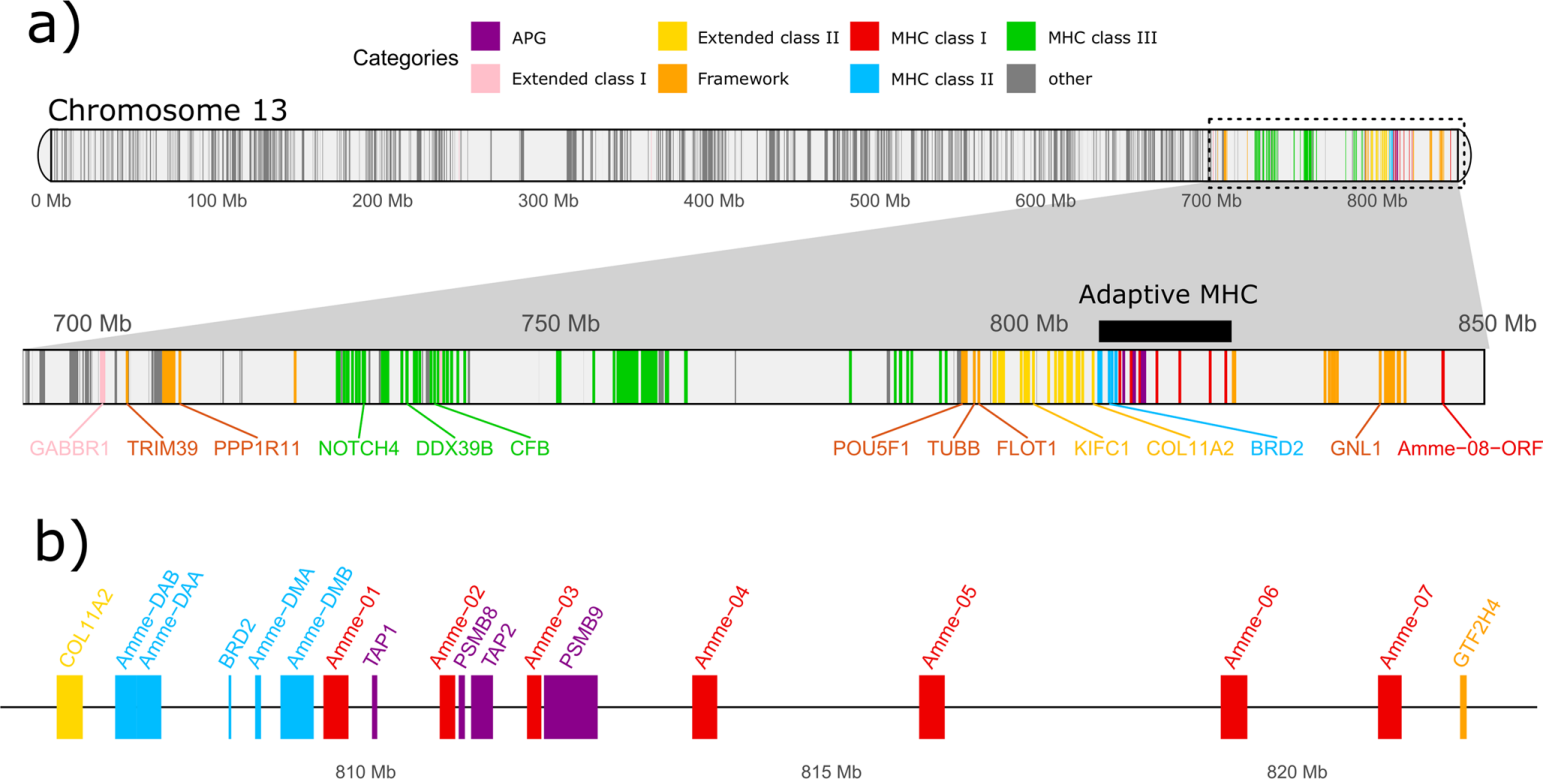
Genomic organization of the MHC region in axolotl. Genes are color-coded according to categories derived from human MHC (see main text). Axolotl’s Class I and Class II regions are closely linked, forming an “adaptive” or “core” MHC region. Antigen processing genes (APG: TAPs and PSMBs) are interspersed among class I genes. Many genes found in human Class III are found clustered together in axolotl, in relative vicinity of the adaptive MHC. Genes found within human extended class II are immediately adjacent to Class II region, and form a tight cluster with conserved synteny. Genes constituting framework of MHC class I region in human were found in various genomic regions: flanking class III genes, and at the chromosome end (past class I genes) – but not interspersed among class I gens (as found in eutherian mammals). (**a**) Visualization of chromosome 13, with a zoomed-in inset encompassing ca. 150 Mb-long region enriched for genes found in human extended MHC. Span of the adaptive MHC is marked with a black bar. (**b**) Gene map of the “adaptive” MHC genes, flanked by extended MHC class II COL11A2 and a framework GTF2H4 genes

As previously reported, the axolotl MHC region is located at the distal end of chromosome 13. However, contrary to earlier interpretations, its structure resembles the typical organization found in non-eutherian tetrapods, featuring a compact, core MHC I/II region (which includes MHC class I and class II genes, as well as APGs) (Fig. 2). Defined in this way, the region is bounded on one end by genes of the extended class II region (as described in the mammalian standard), with *COL11A2* marking its boundary. Immediately following is MHC class II region, with single loci of putative classical MHC II α and β chains (*Amme-DAA* and *Amme-DAB*, respectively). The region also contains the *BRD2* (= *RING3*) gene, as well as a pair of non-classical Class II DM genes (*Amme-DMA* and *Amme-DMB*). Adjacent to these is a cluster of MHC class I genes, interspersed with APGs (*TAP1*, *PSMB8*, *TAP2*, *PSMB9*), forming a complete MHC class I region. Seven MHC class I genes were identified and named in ascending order, according to their genomic location (i.e., from *Amme-01*, closest to the MHC class II region). Last gene, *Amme-07*, is followed by a 18.5 Mb stretch containing several framework genes (sensu Belov et al. 2006), from *GTF2H4* to *DDR1*. An additional gene, *Amme-08-ORF*, is located approximately 3 Mb downstream of *DDR1*, at the end of the chromosome. This gene is partially similar to MHC class I genes, but it contains two extra immunoglobulin-like domains inserted between a putative leader peptide and a putative α1 domain. It is likely a chimeric or fusion gene which, while maintaining an open reading frame, likely lacks typical MHC-associated functions (classical or non-classical), as such functions usually depend on the accessibility of the top, antigen-binding α1–α2 domains. The available transcriptomic data (both RNA-seq and Iso-Seq) contained some sequences with either all domains retained or alternative isoforms, with some putative extracellular domains missing.

Thus, the core MHC region (corresponding to the “adaptive MHC” as defined by Veríssimo et al. 2023), encompassing all likely functional MHC class I and II genes (from *Amme-DAB* to *Amme-07*), spans 13.8 Mb (from 807.3 Mb to 821.1 Mb) (Fig. 2b). In humans, the analogous regions (Class I and II, excluding Class III) span approximately 2.8 Mb jointly. Therefore, the axolotl’s core MHC region is about five times larger compared to the human region, with a genome that is roughly ten times the size of the human genome. We confirmed that this genomic organization was already apparent in the genome used by (Schloissnig et al. 2021); see detailed comparison in Supplementary Note 1 (Supplementary File 1).

Of note, *PSMB10*, the third specialized immunoproteasome unit apart from *PSMB8* and *PSMB9*, typically located on a chromosome different from the MHC region in mammals, was also found on chromosome 13 in the axolotl. There, it is located among class III genes at ~ 726 Mb, and is flanked by putative *C4A* and *EGFL8* orthologs. Similar observation was reported in *Xenopus tropicalis* (Ohta et al. 2006), bowfin (Thompson et al. 2021), and lungfish (Veríssimo et al. 2023).

The number of MHC class I loci described here is higher than the two loci reported by (He et al. 2023), but we note that their analysis was performed using an earlier genome assembly version (the same as used by Schloissnig et al. 2021). At the same time, we report fewer genes than much earlier estimates based on cloning and Sanger sequencing (up to 21 loci) and Southern blot hybridization patterns, which suggested between 10 and 16 MHC-I loci per haplotype (Sammut et al. 1999). The early sequencing-based estimates were likely inflated due to the lack of appropriate correction for PCR and cloning artifacts, a practice that had not yet become standard practice in the field. A careful re-examination of the data supports the presence of up to five loci per individual. This number is much closer to that observed in our study and aligns well with the authors’ own observation of three to five sequence “groups” per individual. Overall, Sammut et al. (1999) defined seven similarity-based sequence groups (termed A-G) among four individuals, which matches the seven consensus sequences we retrieved based on their amplicons.

Analysis of residue conservation in the α1-α2 domains, which are known to anchor peptide termini in classical MHC class I molecules (Kaufman et al. 1994; Saper et al. 1991), revealed significant deviations for *Amme-01* and *Amme-08-ORF* (Table 1). For both loci, all residues involved in C-terminus anchoring are either absent (deletions in alignment) or experienced amino-acid substitutions. For *Amme-08-ORF*, the same applies to N-terminal anchoring (Table 1). Additionally, as noted by Sammut et al. 1999; axolotl sequences universally replaced lysine at position 146 with the charge-equivalent arginine. Moreover, threonine at position 143 is generally not well conserved and is often replaced by serine (which shares similar characteristics, such as an uncharged polar side chain).

**Table 1:** Conservation of class I peptide anchoring residues (based on human MHC consensus). Table organization corresponds to the one presented in (Sammut et al. 1999). Preservation of a conserved residue is marked as “+”, deletion (gap) is denoted with “-”, alternative amino acids substituting conserved residue are shown

		Amme							
		01	02	03	04	05	06	07	08-ORF
N-terminus	Y7	+	+	+	+	G	G	W	+
	Y59	+	+	+	+	+	+	+	E
	Y159	+	+	+	+	+	S	+	D
	Y171	+	+	+	+	+	+	+	L
C-terminus	Y/**R**84	Q	+	+	+	+	+	+	E
	T143	A	S	+	+	+	S	S	W
	K/**R**146	-	+	T	+	+	+	+	-
	W147	-	+	+	+	L	+	+	-
Number of conserved residues		4	7	7	8	6	5	6	1

**Fig. 3:**
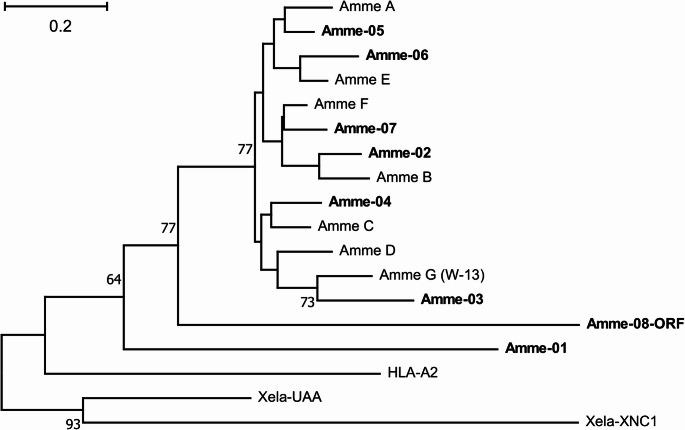
Phylogenetic tree of amino acids sequences of axolotl MHC class I. The genome-derived sequences identified in this study are in bold, named Amme-01-Amme-08-ORF. Consensus sequences (see Methods), representing similarity-based groups reported by (Sammut et al. 1999), are termed Amme A-G. Sequences from other species: Homo sapiens - MHC Ia gene, HLA-A2 (Genbank ID: AAA98727.1), Xenopus laevis - MHC Ia, Xela-UAA (AAF03401.1) and MHC Ib, XNC1 (M58019.1). All sequences were trimmed to match length of Sammut’s consensus sequences. The evolutionary history was inferred by using the Maximum Likelihood method and JTT amino acid evolution model (Jones et al. 1992). The tree with the highest log likelihood (−2969.19) is shown. Bootstrap values > 50 (out of 500 replicates) are shown next to the branches. The tree is drawn to scale, with branch lengths measured in the number of substitutions per site

Due to the overall high similarity of different MHC class I genes and the resulting poor resolution, phylogenetic analysis did not allow an unequivocal assignment of the sequences reported by Sammut et al. (1999) to the loci described in our current analysis (Fig. 3). Furthermore, no clear pattern of grouping between putative Ia and Ib sequences was observed. According to Sammut et al. (1999), who also analyzed patterns of peptide-anchoring residue conservation, their group A likely represented non-classical MHC-Ib sequences (neither T143 nor W147 were conserved), group B exhibited intermediate characteristics (W147 was conserved, and T143 was replaced by S), and groups C–G likely represented polymorphic, classical MHC Ia sequences (both positions were conserved).

**Fig. 4:**
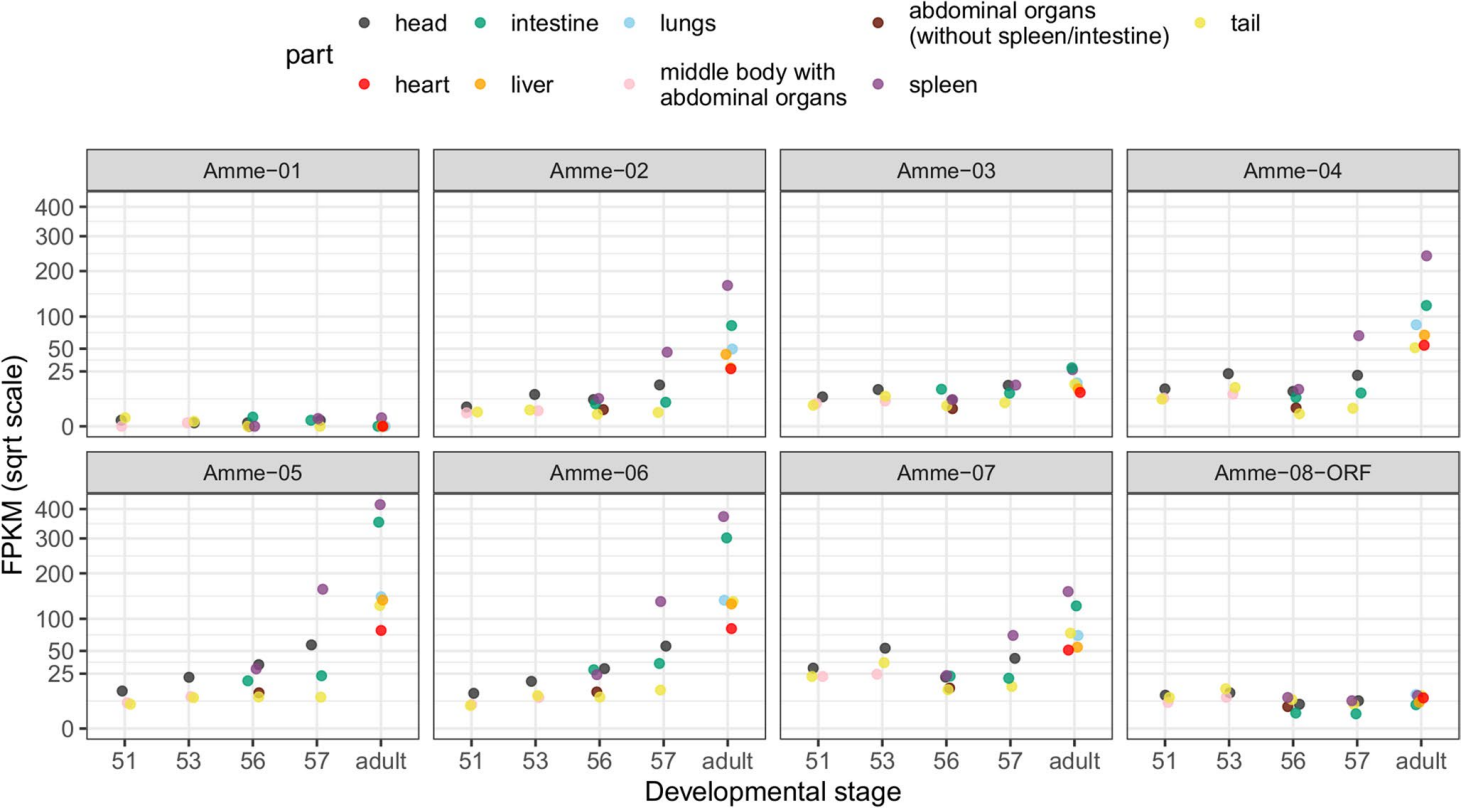
Ontogenetic MHC class I expression profiles. Expression levels were estimated by mapping RNA-seq reads to the genome and are shown as FPKM (y-axis square-root transformed). Tissue of origin is color-coded; one individual per developmental stage was analyzed. Stages are according to Nye et al. (2003)

To further explore the potential functionality and classical versus non-classical status of MHC class I genes, we analyzed their ontogenetic expression profiles. While some expression is detectable as early as stages 53–56 (9–12 weeks post-fertilization; (Nye et al. 2003), substantial splenic expression is only observed at stage 57 (16 weeks) (Fig. 4). With the exception of *Amme-03*, the genes *Amme-02* through *Amme-07* exhibit a consistent pattern, reaching high expression levels. The highest values are found in the adult spleen (FPKM range: 155–416, mean: 245) and intestines, and lower levels are observed in liver, lungs, and skin. *Amme-03* shows markedly lower expression across most tissues and stages, with maximum values just below 30 FPKM in the adult spleen and intestine, suggesting it may represent a class Ib gene despite the preservation of seven anchoring residues. Interestingly, *Amme-05* and *Amme-06*, which preserve only six or five of these residues, respectively, display the highest levels of expression across adult tissues, similar in both spleen and intestine (further suggesting Ib classification). Another gene with six conserved residues, *Amme-07*, exhibits relatively high expression during early larval development (stages 51–53; >25 FPKM), and it continues to rise later on. In contrast, *Amme-01* is essentially non-expressed and likely non-functional. *Amme-08-ORF* exhibits a highly irregular expression pattern, fluctuating between 0 and 13 FPKM across tissues and stages, without consistent trends or tissue specific patterns, suggesting it may lack function and that observed expression reflects transcriptional noise.

Overall, based on anchoring residue conservation, phylogeny, and expression patterns, *Amme-01* and *Amme-08-ORF* are likely non-functional pseudogenes; *Amme-03* may be an MHC Ib gene; *Amme-02*, -*04*, and − *07* are likely MHC Ia genes (though *− 07* could have specialized function at early developmental stages); and *Amme-05* and − *06* may represent either MHC Ia or functionally nuanced MHC Ib genes with high, ubiquitous expression.

The first sequencing-based analysis of axolotl MHC class II expression data (Laurens et al. 2001; Tournefier et al. 1998) suggested the presence of only one, virtually monomorphic MHC class II locus, which contradicted earlier findings based on immunoprecipitation techniques using mammalian cross-reactive MHC class II polyclonal and monoclonal antibodies (Kaufman et al. 1990). Further studies of wild-caught axolotls showed some polymorphism at *DAB*, but no more than two alleles per individual (Richman et al. 2007). In this study, we confirm the presence of a single MHC class II locus that encodes one α and one β chain. Moreover, the amino acid translation of the CDS found in the reference genome is 100% identical to the one reported by Laurens et al. (2001).

Finally, we also investigated a recently described MHC W-category that exhibits mixed features characteristic of both MHC class I and II (Okamura et al. 2021). This category has been identified in various fish lineages, but among tetrapods, it has been found only in certain Urodeles. In this study, we confirmed the presence of an α/β gene pair on the axolotl chromosome 1p, located around 624 Mb. Thus, the putative W-category genes are unlinked to the adaptive MHC region.

## Discussion

Given its structural complexity, high polymorphism, and rapid gene duplication and loss, the MHC region is notoriously challenging to assemble and annotate. At the same time, a well-resolved MHC can attest to the high quality of a newly assembled genome and is often reported to complement genome analyses (Brown et al. 2025; Schloissnig et al. 2021; Session et al. 2016; Thompson et al. 2021). However, if annotation and analysis does not fully consider the evolutionary dynamics of the MHC region, they can mislead conclusions about the processes shaping vertebrate adaptive immunity. The issue is clearly visible in non-eutherian vertebrates, where MHC organization significantly differs from the “gold standard” established for mammals and based on the human MHC region.

For instance, defining the extent of the MHC region in other taxa using the complete set of genes found in the human MHC fails to recognize the region’s primary constituents: the *bona fide* classical MHC genes. While the conserved synteny of certain non-MHC genes within the MHC region can aid in comparative studies, we believe that they should not be used blindly to define the boundaries of the region itself. The MHC class I region in humans, for example, is defined by the genes between *MOG* and *DDX39b* (= *BAT1*). Apart from the *bona fide* MHC class Ia and Ib genes, this region contains non-MHC “framework” genes of well-conserved synteny. These include *TRIM39* orthologs (or paralogues), which are found in the human FW1 segment between the κ and β blocks (Kulski et al. 2002). Since MHC class I genes evolve rapidly and do not maintain 1:1 orthology, even between mammalian orders, relying on these framework genes was crucial for landmark comparative studies between the MHC regions of human and mouse (Amadou 1999). However, the presence of framework genes alone should not be used to define the boundaries of the MHC class I region in non-eutherian taxa. Yet, the “Class I genes” identified by Schloissnig et al. (2021) at the distal end of the putative MHC locus—opposite the class III genes—were, in fact, framework genes (see Supplementary Note 1 for details). Not a single MHC Ia, Ib or MHC-like gene was mapped there in Schloissnig et al. (2021), nor could one be found in our current annotation (Fig. 2). The misassignment of these genes led to an inflated estimate of the axolotl MHC region at 100 Mb in Schloissnig et al. (2021) compared to the 13.8 Mb reported here.

Crucially, the salamander MHC does not exhibit the split class I arrangement characteristic of eutherian mammals (Fig. 1). Therefore, the suggestion placed by Schloissnig et al. (2021) that “the presence of the class III region between class I and II is a shared ancient arrangement in the common ancestor of modern amphibians and mammals” represents a clear misinterpretation. In the axolotl, not a single bona fide MHC class I gene is separated from the class II by class III region. Meanwhile, the presence of certain framework genes in proximity to the core MHC has been well-established in many non-mammalian species (Ohta et al., 2015). A scenario in which class I genes were translocated into the framework region in the common ancestor of eutherian mammals has been suggested before (Belov et al. 2006).

However, it is equally important, to adhere to concepts and nomenclature standards derived from the human MHC locus when necessary, as disregarding them may hinder evolutionary interpretation. A relevant example comes from a recent report on MHC organization across several amphibian species, including the urodele *Ambystoma mexicanum* and *Pleurodeles waltl*, as well as the caecilian *Microcaecilia unicolor* (He et al. 2023). While the overall conclusions presented in that study are broadly consistent with our findings, highlighting a core MHC I/II structure with APGs interspersed among MHC class I genes, the analysis fell short of properly identifying synteny and applying standard MHC genomic nomenclature. Specifically, the terms “extended class I” and “extended class II” were used rather loosely, often in a literal sense to describe genomic regions flanking the identified class I and class II gene clusters. While this approach may have some merit, it deviates from the terminology established in the mammalian literature. In the case of the so-called “extended class II” region, the gene content largely corresponded to that in mammals, mitigating confusion. However, none of the genes identified in their “extended class I” region matched what is canonically defined as such in mammalian models. This is particularly evident in the caecilians. There, the genes labeled as belonging to the “extended class I” region actually correspond to class III genes in the mammalian MHC (*NOTCH*, *PBX2*, *RNF5*, *C4*, *FKBPL*, and *DXO*), followed by framework genes like *GNL1*, *ABCF1*, *DHX16*, and *FLOT1*. This gene order is consistent with the ancestral tetrapod MHC structure proposed by Belov et al. (2006). In the axolotl, the overall MHC structure described by He et al. (2023) is broadly similar to the one we report here, though notably smaller: their core MHC spans 6.2 Mb, compared to 13.8 Mb in our annotation. This discrepancy is primarily due to the lower number of MHC class I loci identified in their study (two versus seven in this study), which was based on a different assembly. Additionally, the genes labeled as part of the “extended class I” region in that analysis correspond, as shown in our current annotation, either to framework genes directly adjacent to the Class I/APG cluster, or to a mix of extended class II and framework genes placed beyond the extended class II defined by the authors. This indicates presence of lineage-specific rearrangements in axolotl, such as inversions and translocations, relative to the ancestral MHC organization (Figs. 1 and 2).

Overall, greater precision in the nomenclature used by He et al. (2023) would have helped to identify the syntenic relationships. Reanalysis employing the standard, human-derived gene classification enables two key insights. First, the gene cluster found within the so-called extended class II region appears to have been adjacent to the classical class II genes already in early tetrapods. This arrangement may even reflect an ancestral genomic configuration, dating back to basal sarcopterygians, as suggested by lungfish data (Veríssimo et al. 2023). Second, the data strongly support the existence of a cluster of framework genes, situated next to the class III region and opposite the core MHC Class I/II region in the ancestral tetrapod. The data also highlight the co-occurrence of many genes found in the mammalian Class III region in this early-branching tetrapod. These observations align with the model proposed by Belov et al. (2006) but also highlight lineage-specific genomic rearrangements—such as inversions or translocations—in various urodele species. Our current analysis reveals a separation of Class III from core ClassI/II region by extended class II, an unusual organization among studied tetrapods (Fig. 1). Importantly, despite these rearrangements, the fundamental architecture of a compact, core MHC I/II region remains conserved.

The emerging genomic context is significant not only for reconstructing the ancestral state of the MHC region, but also for understanding the evolutionary trade-offs that shape adaptive immune capacities in non-mammalian vertebrates. The physical organization and proximity of key gene groups within the MHC region, in particular, can inform hypotheses about co-evolutionary dynamics. The tight linkage of classical MHC class I genes and APGs within the core MHC has been proposed to facilitate the evolution of matching specificities between MHC peptide-binding grooves and the peptides generated and transported by APGs. In turn, this co-evolutionary relationship has been suggested to constrain the duplication and diversification of MHC class Ia genes in non-mammalian vertebrates. In contrast, in mammals breaking of the physical linkage between Ia and APGs by Class III supposedly released class Ia genes from the co-evolutionary pressures imposed by APGs and thereby enabled the expansion of MHC class Ia into multi-gene families (Nonaka et al. 1997; Ohta et al., 2015). However, the situation appears more complex. Increasing evidence suggests that MHC class I gene expansion has also occurred in several non-mammalian vertebrate lineages, although whether such expansion took place within the core MHC I/II region and near the APGs is still debated. For example, a detailed analysis of MHC organization in cartilaginous fish revealed the maintenance of linkage between MHC class I and class II genes on a single chromosome in early-branching gnathostomes (Veríssimo et al. 2023). In this case, both MHC Ia and MHC Ib genes are present, but Ib genes are embedded among numerous zinc finger protein genes, which were proposed to inhibit recombination with the tightly linked Ia gene. Similarly, in the opossum—a non-eutherian mammal with a more ancestral MHC architecture—tight linkage with APGs has not prohibited the duplication of MHC-I genes, though the majority of these are believed to be MHC Ib, with only one suggested to be MHC Ia (Belov et al. 2006).

In our current analysis, we confirm the presence of a multi-gene MHC class I family that is tightly linked to APGs in the axolotl. While the classical or non-classical nature of these genes has yet to be fully established, our findings demonstrate that gene expansion is possible even within a tightly linked core MHC region. Nevertheless, the extent to which co-evolution with APGs constrains or facilitates such expansions remains an open question. Notably, a broad comparative study of urodele amphibians suggested that co-evolution with APGs may persist even in the presence of expanded MHC class I families. This idea was supported by the observed correlations between MHC class I and APGs diversity metrics (Palomar et al. 2021), which imply that coordinated diversification may still be occurring. That said, the extent of gene duplication may be limited by such arrangement, and separation of APGs and MHC class I could still promote within-individual diversification. Support for this scenario comes from the extremely duplicated MHC class I genes in passerine birds, where TAP genes are mostly unlinked to the MHC class I (He et al. 2022; Westerdahl et al. 2022).

Interestingly, MHC class I genes show overall low expression throughout larval development, with some genes gradually increasing towards full larval maturity (stage 57, here ~ 16 weeks post-fertilization), and reaching high levels in adulthood. This pattern is broadly consistent with earlier Northern blot observations, in which MHC class I mRNA was first detectable at the hatching stage, remained low until about two months (though detectable in the spleen), and increased markedly by three months, reaching adult levels around four months of age (Tournefier et al. 1998). This developmental pattern is somewhat reminiscent of that observed in *Xenopus*, where larval MHC Ia expression is low and metamorphosis represents a clear demarcation point after which ubiquitous MHC Ia expression and robust adaptive immune responses appear. Nonetheless, *Xenopus* exhibits adaptive immune activity earlier in development, with circulating CD8⁺ T cells present already in tadpoles, restricted by some MHC Ib molecules (Edholm et al. 2013, 2014). In contrast, axolotl development is slower, and the species does not naturally undergo metamorphosis; the absence of a clear developmental transition complicates direct comparisons of developmental trajectories. Whether any of the axolotl class I genes described here perform analogous functions to *Xenopus* MHC Ib genes remains uncertain, although a relatively high expression of *Amme-07* at early larval stages might indicate a similar role.

## Conclusions

Our findings highlight the critical importance of recognizing the unique features of complex genomic regions, such as the MHC, to ensure accurate annotation. This is essential for producing reliable insights that can inform diverse fields, from immunology to evolutionary biology. Misinterpretations, especially those rooted in incomplete or inconsistent annotation, can easily propagate across studies and disciplines, leading to skewed conclusions. A consistent and well-defined nomenclature is key to avoiding these pitfalls. To that end, we advocate for the broader adoption of the gene categories proposed by Belov et al., as they are well-suited for describing MHC organization across both eutherian and non-eutherian vertebrates while maintaining clear correspondence with the gold standard of the human MHC nomenclature. We particularly recommend distinguishing the antigen-processing gene (APG) category, rather than referring to these genes as part of the “class II region”, which is common in mammalian literature. We also strongly support using the “framework” category to describe non-MHC genes of conserved synteny interspersed within the MHC class I region in mammals, but found in different genomic positions in other vertebrate groups. Finally, the question of what constitutes MHC region, particularly the nature of Class III and the role of framework genes (Amadou 1999; Kaufman et al., 1995; Powis et al., 1995), have long been debated. Yet, we argue that, in any species, it should be defined primarily by the span of *bona fide* MHC class I and class II genes. In essence, MHC region should be delineated by the location of genes directly involved in antigen presentation, rather than by the presence of associated or merely syntenic elements.

## Supplementary Information

Below is the link to the electronic supplementary material.

Supplementary File 1 (PDF 272 KB)

Supplementary File 2 (FASTA 12.7 KB)

Supplementary File 3 (GFF84.6 KB)

Supplementary File 4 (FASTA 1001 bytes)

## Data Availability

Additional materials, including Supplementary Notes, are available as Supplementary Files associated with this manuscript. Raw RNAseq sequence data have been deposited in the European Nucleotide Archive under the ENA Research Project accession PRJEB96841.
