# Supplementary material for: Compact genomic architecture of the axolotl MHC region: setting the record straight
Source: Immunogenetics. 2025 Nov 15;77(1):33. doi: 10.1007/s00251-025-01391-x (PMC12619710; doi:10.1007/s00251-025-01391-x)
Supplement: Supplementary file 1 — (PDF 272 KB) [file 251_2025_1391_MOESM2_ESM.pdf]

Supplementary Note 1

Differences between AmexG_v6.0-DD and UKY_AmexF1_1 assemblies

Genome assemblies and key statistics

The MHC region annotation presented in this work was based on the latest available chromosome-scale genome assembly of the axolotl: UKY_AmexF1_1 (GCA_040938575.1). This assembly is distinct from AmexG_v6.0-DD (GCA_002915635.3), which was presented by Schloissnig et al. (2021)[1] and used in their description of the MHC region accompanying the publication of that assembly. UKY_AmexF1_1 is a maternal pseudohaplotype generated from the genome sequence of an F1 female, the offspring of a cross between a female *Ambystoma mexicanum* and a male *A. tigrinum*. AmexG_v6.0-DD is based on the genome of a male individual from strain DD151. While the total genome sizes and scaffold N50 values are broadly comparable between the AmexG_v6.0-DD and UKY_AmexF1_1 (28.2 Gb vs. 29.1 Gb; N50: 1.2 Gb vs. 1.5 Gb, respectively), the contig N50 is substantially higher for the latter (218 kb vs. 23.1 Mb). The improved quality of the UKY_AmexF1_1 assembly is also evident in the reported numbers of scaffolds (27,157 vs. 220) and contigs (211,437 vs. 2,315).

Differences between assemblies in the MHC region

To examine how the broader region identified as the MHC differs between the assembly used by Schloissnig et al. (2021) and the UKY_AmexF1_1 assembly used here, we identified syntenic blocks between the two assemblies within the genomic region spanning from the start of *GABBR1* to the end of chromosome 13 (exact coordinates: AmexG_v6.0-DD - chr13q:377,763,360–483,343,317; UKY_AmexF1_1 - NC_090938.1:700,792,965–848,590,089). Syntenic blocks were identified using ntSynt[2] under the assumption of ~1% sequence divergence between assemblies. The comparison revealed that, while some local differences are visible, the overall organization of the genomic region encompassing the MHC is broadly similar (Figure S1). No major rearrangements were observed that could account for the reported disparity in the estimated size of the MHC region. In fact, the overall region is larger in the UKY_AmexF1_1 assembly.

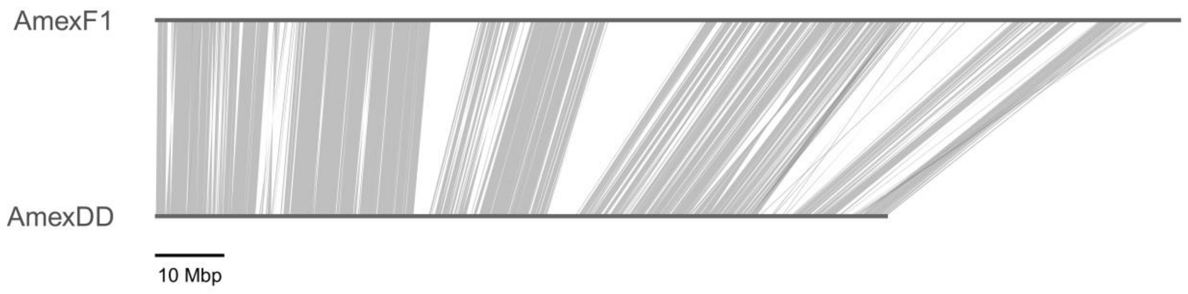

Figure S1. Synteny blocks of minimum length 100 kb identified between the UKY_AmexF1_1 assembly (Amex F1, used in this study) and the AmexG_v6.0-DD assembly from Schloissnig et al. (2021) (Amex DD). The displayed region spans from the start of *GABBR1* to the end of chromosome 13.

Subsequently, we reanalyzed the region utilizing the annotations provided by Schloissnig et al. (2021) (their Supplementary Data S02). We present the gene content as originally reported by Schloissnig et al. (2021), but color-coded according to the categories used consistently throughout

our manuscript (i.e., extended Class I and II, framework genes, APGs, and MHC Class I, II, and III) - Figure S2. From this presentation it becomes evident that the confusion regarding the MHC region size arises entirely from the interpretation of annotated genes and their assignment to the Class I category, rather than from issues with the assembly itself.

Similar to UKY_AmexF1_1 (Figure 2 of the main text), the AmexG_v6.0-DD assembly also contains a cluster of Framework genes adjacent to the Class III region, on the opposite side of the core/adaptive MHC. These were misinterpreted and labeled as Class I in Schloissnig et al. (2021), which led to their suggestion of a split Class I arrangement. Importantly, the length of what can be unambiguously defined as the core/adaptive MHC in the files from Schloissnig et al. (2021) is comparable to our annotation of UKY_AmexF1_1 - approximately 16 Mb. All genes that can be confidently assigned to bona fide Class I or Class II categories (whether classical or non-classical) are located within this core MHC region, and no bona fide Class I genes were annotated on the opposite side of the MHC Class III region (Figure S2), where only Framework genes are found.

Additionally, while the core region in AmexG_v6.0-DD appears to contain somewhat more annotated genes and shows some differences in gene order (compare Figure S2b with Figure 2b in the main text), some of these annotations are likely incomplete and/or misassembled. This interpretation is supported by the more detailed analysis of He et al. (2023)[3], who used the same genome assembly as Schloissnig et al. (2021) and reported fewer complete genes within this region.

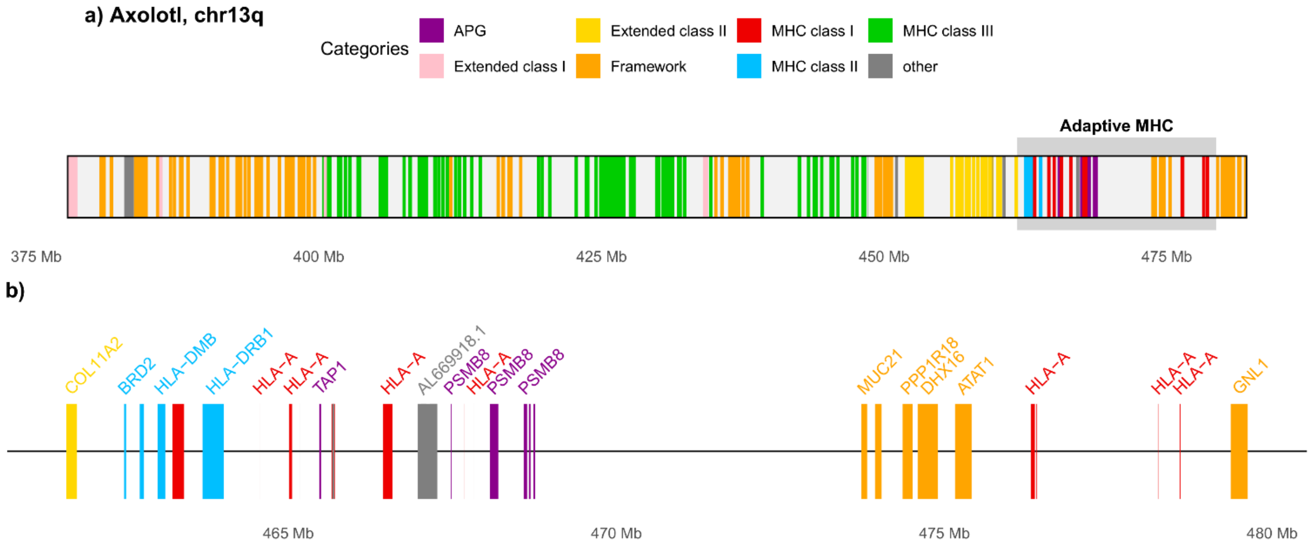

Figure S2. Genomic organization of the MHC region in axolotl as annotated in Schlossing et al. 2021. Figure is based on Supplementary Data S02 associated with the original publication, but genes were assigned categories and color-coded according to categories maintained throughout the main text. Axolotl's Class I and Class II regions are closely linked, forming an "adaptive" or "core" MHC region. a) Visualization of ca. 100 Mb-long region enriched for genes found in human extended MHC. The adaptive MHC is marked with a grey box. b) Gene map of the adaptive/core MHC genes, flanked by extended MHC class II *COL11A2* and Framework *GNL1* gene.

Supplementary Note 2

MHC class I expression analysis

Animal husbandry

Axolotls were obtained from the colony at the Technical University of Dresden, Centre for Regenerative Therapies Dresden, Germany (registration number: DD24.1-5131/3346/10). Animals were housed in aquaria placed in a ventilated room maintained at approximately 50% humidity and a temperature of 19 °C, under a 12-hour light/12-hour dark cycle. About 50% of the water was replaced three times per week. Animals were kept in separate aquaria until mating. After egg laying, the eggs were transferred to new containers. Upon hatching, larvae were fed *ad libitum* with Artemia nauplii, and after approximately three weeks, with live chironomid larvae.

Tissue collection

Larvae at stages 51, 53, 56, and 57 (staged according to Nye et al., 2003)[4] were euthanized using MS-222 and preserved in RNAlater (Table S1). Depending on size, individuals were cut into two or three parts (head, middle, tail), and/or individual organs were preserved separately. Additionally, one adult female (2.5 years old) was sacrificed by MS-222 overdose, and the heart, intestine (middle segment), liver, lungs, spleen, and tail tissue were preserved in RNAlater. A complete list of samples is provided in Table S1. All experimental procedures were conducted in accordance with Resolution No. 13/2023 (January 12, 2023) issued by the Second Local Ethics Committee for Animal Experimentation in Kraków.

Table S1. List of animals used for expression analysis.

ID	*stage*	*mother ID*	*age*
25424	51	25245	6 weeks
25465	53	25245	9 weeks
25479	56	25245	12 weeks
25481	57	25245	16 weeks
25245	adult	NA	2.5 years

RNA extraction and Illumina RNAseq

Total RNA was extracted from RNAlater-preserved tissues using either RNAzol® RT (Sigma) or RNeasy (Qiagen) kit. RNA-seq libraries were prepared using Novogene’s Plant and Animal Eukaryotic Strand-Specific mRNA protocol (WOBI) and sequenced (2 × 150 bp) on the Illumina NovaSeq platform by Novogene.

Supplementary References

1. Schloissnig S, Kawaguchi A, Nowoshilow S, et al. The giant axolotl genome uncovers the evolution, scaling, and transcriptional control of complex gene loci. *Proc Natl Acad Sci U S A*. 2021;118(15). doi:10.1073/PNAS.2017176118/-/DCSUPPLEMENTAL

2. Coombe L, Kazemi P, Wong J, Birol I, Warren RL, Smith M. Multi-genome synteny detection using minimizer graph mappings. *bioRxiv*. February 2024:2024.02.07.579356. doi:10.1101/2024.02.07.579356

3. He K, Babik W, Majda M, Minias P. MHC Architecture in Amphibians—Ancestral Reconstruction, Gene Rearrangements, and Duplication Patterns. *Genome Biol Evol*. 2023;15(5). doi:10.1093/GBE/EVAD079,

4. Nye HLD, Cameron JA, Chernoff EAG, Stocum DL. Extending the table of stages of normal development of the axolotl: Limb development. *Dev Dyn*. 2003;226(3):555-560. doi:10.1002/dvdy.10237
